# Assessing the Impacts of Adaptation to Native‐Range Habitats and Contemporary Founder Effects on Genetic Diversity in an Invasive Fish

**DOI:** 10.1111/eva.70006

**Published:** 2024-10-04

**Authors:** Thaïs A. Bernos, Zdenek Lajbner, Petr Kotlík, Jacklyn M. Hill, Silvia Marková, Jonah Yick, Nicholas E. Mandrak, Ken M. Jeffries

**Affiliations:** ^1^ Department of Ecology and Evolutionary Biology University of Toronto Toronto Ontario Canada; ^2^ Department of Biological Sciences University of Toronto Scarborough Toronto Ontario Canada; ^3^ Physics and Biology Unit Okinawa Institute of Science and Technology Graduate University Okinawa Japan; ^4^ Laboratory of Molecular Ecology Institute of Animal Physiology and Genetics, Czech Academy of Science Prague Czechia; ^5^ Maurice Lamontagne Institute, Fisheries and Oceans Canada Mont‐Joli Quebec Canada; ^6^ Inland Fisheries Service New Norfolk Tasmania Australia; ^7^ Department of Biological Sciences University of Manitoba Winnipeg Manitoba Canada

**Keywords:** freshwater fish, genetic structure, genomics, invasion dynamics, non‐native species, population diversity

## Abstract

Species invading non‐native habitats can cause irreversible environmental damage and economic harm. Yet, how introduced species become widespread invaders remains poorly understood. Adaptation within native‐range habitats and rapid adaptation to new environments may both influence invasion success. Here, we examine these hypotheses using 7058 SNPs from 36 native, 40 introduced and 19 farmed populations of tench, a fish native to Eurasia. We examined genetic structure among these populations and accounted for long‐term evolutionary history within the native range to assess whether introduced populations exhibited lower genetic diversity than native populations. Subsequent to infer genotype–environment correlations within native‐range habitats, we assessed whether adaptation to native environments may have shaped the success of some introduced populations. At the broad scale, two glacial refugia contributed to the ancestry and genomic diversity of tench. However, native, introduced and farmed populations of admixed origin exhibited up to 10‐fold more genetic diversity (i.e., observed heterozygosity, expected heterozygosity and allelic richness) compared to populations with predominantly single‐source ancestry. The effects of introduction to a new location were also apparent as introduced populations exhibited fewer private alleles (mean = 9.9 and 18.9 private alleles in introduced and native populations, respectively) and higher population‐specific *Fst* compared to native populations, highlighting their distinctiveness relative to the pool of allelic frequencies across tench populations. Finally, introduced populations with varying levels of genetic variation and similar genetic compositions have become established and persisted under strikingly different climatic and ecological conditions. Our results suggest that lack of prior adaptation and low genetic variation may not consistently hinder the success of introduced populations for species with a demonstrated ability to expand their native range.

## 1 Introduction

Species invading regions outside of their native range can irreversibly alter trophic structures, outcompete native species, introduce pathogens and disease and may incur billions of dollars in management‐related costs (Castilla et al. 2005; Pimentel et al. 2000; Sax et al. 2007; Shackleton, Shackleton, and Kull 2019). As the number and the geographic scale of human‐assisted introductions increase rapidly (Gallardo 2016), understanding the factors influencing invasion success is central to improving management efforts. Previous studies suggested that invasion success can be facilitated by species' tolerance to environmental stressors (Bates et al. 2013; Lenz et al. 2011), specific life‐history traits (Capellini, Allen, and Sally 2015; Chapple, Simmonds, and Wong 2012) and high phenotypic plasticity (Komoroske et al. 2021; Wellband and Heath 2017). Additionally, evolutionary processes including rapid adaptation to novel environments and adaptation within the native range may also facilitate population establishment and subsequent expansion within novel habitats (Baker and Stebbins 1964; Hufbauer et al. 2012; Lee 2002).

Invasiveness may be facilitated by rapid adaptation following species' introduction (Baker and Stebbins 1964; Lee 2002). Selection likely operates on standing genetic variation because, in small populations, the supply of de novo mutations is limited (Dlugosch and Parker 2008; Prentis et al. 2008). Founder events, inbreeding and genetic drift are expected to reduce genetic variation—including adaptive alleles and balanced polymorphism—in introduced populations (Chakraborty and Nei 1977; Grapputo et al. 2005; Nei, Maruyama, and Chakraborty 1975; Tsutsui et al. 2000). Therefore, the ability of invasive populations to thrive in novel environments is often deemed paradoxical. However, the general applicability of the “genetic paradox of invasive species” (Allendorf and Lundquist 2003) has been challenged, as introduced populations do not always exhibit low genetic variation (Bossdorf et al. 2005; Dlugosch and Parker 2008; Kolbe et al. 2004; Jaspers et al. 2021; Uller and Leimu 2011). Hence, the mechanisms by which demographic and selective factors influence patterns of genetic diversity to either facilitate or impede invasion success remain subjects of debate.

Adaptive divergence within the native range can also enhance invasion success (Bossdorf, Lipowsky, and Prati 2008; Hufbauer et al. 2012). For example, adaptation to low winter flow within the native Pacific Coast range of rainbow trout (*Oncorhynchus mykiss*) might have facilitated its invasiveness in similar environments of the non‐native southern Appalachians (Fausch 2008), where the invasive fish supplanted native brook trout (*Salvelinus fontinalis*). Other forms of prior adaptation being beneficial in non‐native ranges may include tolerance to environmental stressors (Bates et al. 2013; Lenz et al. 2011), specific life‐history traits (Capellini, Allen, and Sally 2015; Chapple, Simmonds, and Wong 2012) or phenotypic plasticity (Komoroske et al. 2021; Wellband and Heath 2017). While prior adaptation could promote invasiveness, this mechanism has rarely been investigated in genetic studies of invasive species because it requires extensive sampling and identification of local adaptation in both native and introduced populations. Studies examining species throughout both native and introduced ranges remain rare despite the valuable insights they offer into the adaptive mechanisms underlying invasion success (Colautti et al. 2017).

Here, we examined the evolutionary mechanisms facilitating invasiveness in the tench (*Tinca tinca L*.), a fish native to Eurasia that has been reportedly introduced to all continents except Antarctica for aquaculture and recreational fishing purposes (Delpero and Volpato 2022). Tench is invasive in several regions (e.g., Canada, United States, New Zealand and Australia) where it has been linked to changes in water quality, disruption of native communities and introduction of pathogens (reviewed in Avlijas, Ricciardi, and Mandrak 2017; Rowe 2004). The species possesses some characteristics favouring invasiveness, including broad environmental tolerance and high fecundity (Avlijas, Ricciardi, and Mandrak 2017). Earlier genetic studies revealed that tench was characterized by two deeply divergent phylogroups (Lajbner et al. 2010; Lajbner, Linhart, and Kotlík 2011); population sampled from the extremities of the native range were assigned to distinct phylogroups corresponding to glacial refugia while central populations showed mixed ancestry. These findings suggest that: (1) the process of expansion from glacial refugia influenced the distribution of genetic variation among native source populations and (2) each phylogroup could be adapted to different parts of the range. Apart from two recent studies employing next‐generation sequencing technologies to study invasive and farmed populations (Bernos et al. 2023; Kumar et al. 2019), previous population genetic research on tench relied on smaller numbers of mitochondrial and/or nuclear markers (Al Fatle et al. 2022; Karaiskou et al. 2020; Kohlmann et al. 2010; Lajbner et al. 2010; Lajbner, Linhart, and Kotlík 2011; Lajbner and Kotlik 2011; Lo Presti et al. 2010). Therefore, the genome‐wide population structure and genetic diversity of native and introduced tench populations remain poorly understood.

Much of the previous literature examining the evolutionary mechanisms facilitating invasiveness relied on a small number of genetic markers or is based on a limited number of sampled locations. The current study built on these earlier insights provided by these previous research efforts by: (1) disentangling the consequences of long‐term historical segregations between native source populations and contemporary introductions on range‐wide patterns of genetic diversity; (2) screening for genetic variants associated with local climates (gene–environments relationships) in the native range; and (3) extrapolating gene–environment relationships to the introduced range to evaluate the role of prior adaptation in facilitating invasiveness. Using data from 7958 single nucleotide polymorphisms (SNPs) identified by genotyping by sequencing (GBS; Poland et al. 2012) across 95 tench populations, including 36 native, 40 introduced and 19 farmed populations, we examined the relative contributions of contemporary introductions and prior adaptation to shaping the species' adaptive potential and driving invasiveness.

## 2 Methods

### 2.1 Samples and Genotyping by Sequencing

**FIGURE 1:**
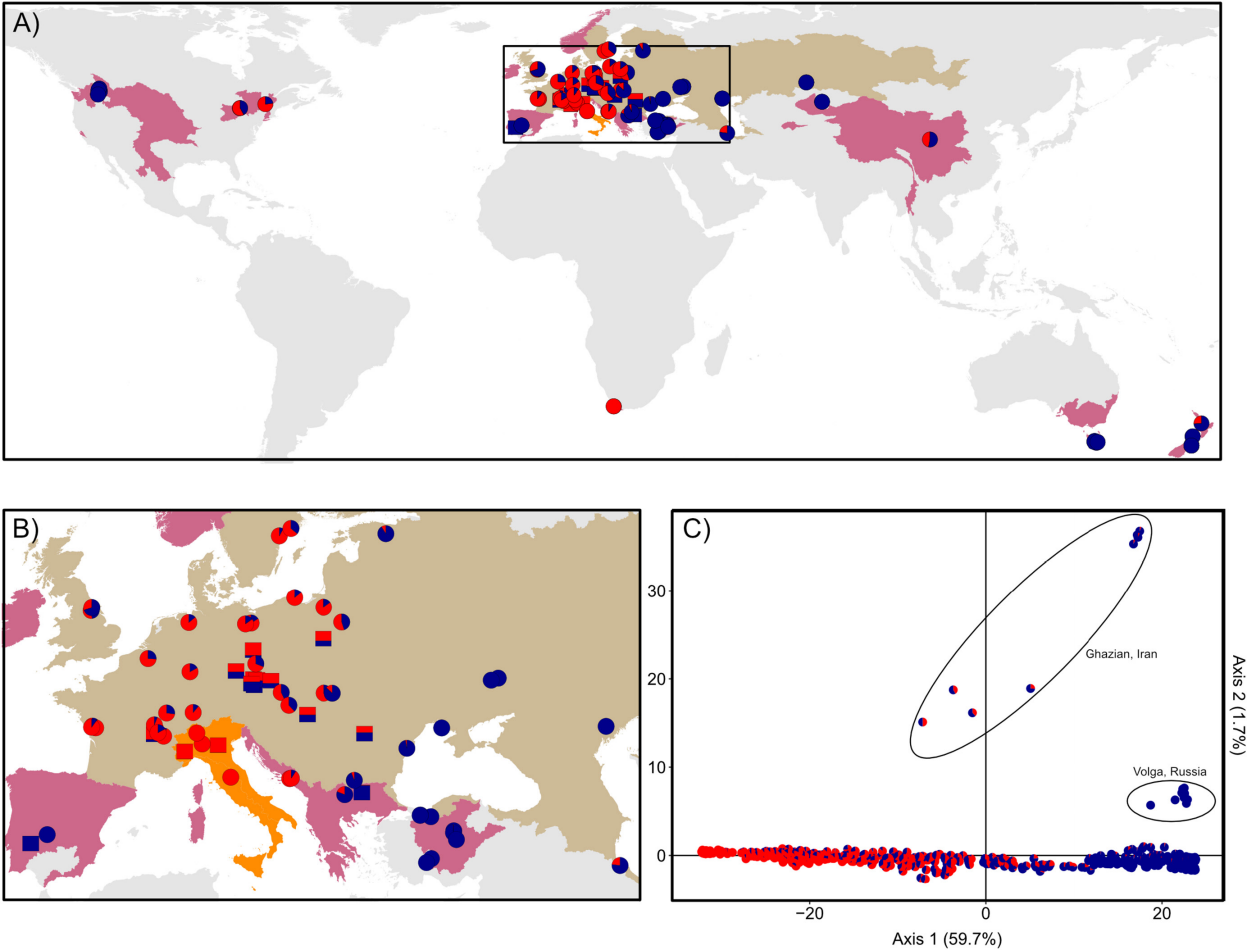
Distribution of tench (*Tinca tinca*) populations used in this study. Figures showing the geographic distribution (A, B) and principal component analysis (PCA) plot (C) for two ancestral genetic clusters (*K* = 2) identified by Admixture. In (A, B), colours of the pie chart (wild populations) and bar plot (farmed populations) denote the ancestral composition of each tench population (Eastern cluster = blue and Western cluster = red) across the native (olive), introduced (violet) and cryptogenic (i.e., uncertain) range (orange). In (C), pie charts denote the ancestral composition of each individual.

We used fin‐clip samples from 768 individuals collected from 39 native populations (defined as a group of organisms inhabiting the same area), 14 introduced populations and 19 farmed populations (Figure 1). These samples included 550 individuals previously genotyped using one mitochondrial and three nuclear loci (Lajbner, Linhart, and Kotlík 2011) and 218 newly added individuals. The introduced populations examined were introduced as early as 1860 (e.g., New Zealand: Rowe 2004) and as recently as 1986 (e.g., Canada; Dumont et al. 2002) and included populations managed as threats to native ecosystems (e.g., Canada, United States and New Zealand). Nuclear DNA was isolated with the E‐Z 96 Tissue DNA Kit (Omega BIO‐TEK Inc, Norcross, GA, USA), quantified and normalized at the sequencing Institute of Integrative Biology and Systems (Québec City, QC, Canada). Genotyping‐by‐sequencing libraries were prepared using two restriction enzymes (Pst1 and Msp1). Paired‐read sequencing was performed on a NovaSeq 6000 PE100 at the Centre d'expertise et de services Génome Québec (Montréal, QC, Canada).

### 2.2 Bioinformatics

We used the STACKS v2.3 pipeline (Catchen et al. 2013) to filter raw reads, assemble reads into loci, identify variants and score genotypes using *de novo* assembly as there is no high‐quality reference genome for tench or closely related species. To identify the parameters optimizing the *de novo* assembly of loci, we first ran STACKS on 20 samples to test 16 combinations of parameters including the ustack parameters m (the minimum depth of coverage required to create a stack) from 1 to 6, and M (the number of mismatches allowed between stacks to merge them into a putative locus) from 0 to 7; and the cstack parameter *n* (the number of mismatches allowed during the construction of the catalogue) from M − 1 to M + 1 (Paris, Stevens, and Catchen 2017). We visualized the effects of M, m and *n* values on summary statistics (i.e., average sample depth, number of SNPs retained and number of loci retained) using RADstackshelpR (DeRaad 2021) (Figure S1). Then, we ran STACKS on all samples using the identified parameter values, including the following ustacks (*m* = 3, *M* = 2, model type = bounded and bound high = 0.05) and cstacks (*n* = 3) parameters. For catalogue assembly, we picked two samples with high coverage per location to minimize the noise (Rochette and Catchen 2017). We processed the output file using VCFtools v0.1.16 (Danecek et al. 2011), applying loose population filters (maximum proportion of missing data [−‐max‐missing] = 30%, minimum minor allele count [−‐min‐mac] = 2). Additionally, we removed low‐confidence SNP calls by recoding SNPs with <3 reads as missing (minDP = 3), excluded loci with a mean genotype depth across individuals <15 (min–mean DP = 15) and filtered out SNPs genotyped in <80% of individuals (miss‐sites = 80) and individuals genotyped in <50% of loci (miss‐ind = 50), respectively. Finally, we used the population module in STACKS to retain a single SNP per locus (choosing the first SNP for loci with more than one SNP; −‐write_single_snp) and exported the final data in vcf format.

### 2.3 Inferring Population Structure and Evolutionary Relationships

We examined population structure using complementary model‐based (ADMIXTURE, Alexander, Novembre, and Lange 2009) and multivariate (principal components analysis, PCA) approaches. As ADMIXTURE is less accurate in situations where many populations are sampled and sample sizes are uneven between sampled locations (i.e., highly diverged populations with few individuals may not be assigned their own cluster; Puechmaille 2016), we tested *K* values between 1 and 20 at two hierarchical levels: all samples together (broad scale) and separately within farmed, native and introduced populations (fine scale). To determine the “best‐fit” *K* for each dataset, we used the ADMIXTURE 10‐fold cross‐validation procedure. We examined the convergence of ADMIXTURE results at the broad, spatial scale with the patterns of genetic variation inferred visually from an individual‐based PCA implemented in the R package ade4 (Dray and Dufour 2007).

To further understand the evolutionary context of tench population structure, we used a method overlaying pairwise *Fst* and population‐specific *Fst* (Kitada, Nakamichi, and Kishino 2021). While large pairwise *Fst* indicates strong population differentiation due to population structure, population‐specific *Fst* provides an estimate of the divergence of a population from the ancestral population (Kitada, Nakamichi, and Kishino 2021). First, we used the function snpgdsFst (i.e., fixation index) from SNPRelate (Zheng et al. 2012) to compute pairwise *Fst* values between each population pair and the function betas (i.e., estimates of individual inbreeding coefficients) from hierfstat (Goudet and Jombart 2022) to calculate population‐specific *Fst*. Second, we produced an unrooted neighbour‐joining tree using the nj (i.e., neighbour‐joining) in ape (Paradis and Schliep 2019) and performed a multidimensional scaling (MDS) analysis on the pairwise *Fst* matrix. Then, we placed this population structure analysis within an evolutionary context by colouring populations based on the magnitude of their population‐specific *Fst*.

### 2.4 Contrasting Genetic Diversity and Differentiation Among Introduced, Native and Farmed Tench

To assess whether genetic diversity consistently differed among introduced, native and farmed populations, we calculated observed heterozygosity (*Ho*), expected heterozygosity (*He*), nucleotide diversity (*Pi*) and number of private alleles (*Pa*) using STACKS. Subsequently, we investigated differences using linear models (*Ho*, *He* and *Pi*) and a general linear model with a Poisson distribution (*Pa*). These models incorporated population status (introduced, native or farmed) and a polynomial term (ancestry proportion to the Western cluster^2^) to account for potential curved relationships between genetic diversity metrics and admixture proportion to two genetic clusters inferred by admixture. The significance of the fixed effects was tested via likelihood ratio tests in a model including population status (introduced, native or farmed) and admixture proportion as fixed effects.

### 2.5 Investigating Genotype–Environment Correlations

To characterize the environmental conditions at each sampling location for subsequent analyses (latent factor mixed model [LFMM] and partial redundancy analysis [pRDA]), we selected four bioclimatic variables at a 10‐arcminute resolution from WorldClim (Fick and Hijmans 2017) that are potentially relevant for the life history of freshwater fish. Variables included minimum and maximum temperature of the coldest and the warmest months (a potential indicator of thermal stress; Neuheimer et al. 2011), mean annual temperature and temperature seasonality (both influence fish physiology and metabolism; Fry 1971) and annual precipitation (which affects water chemistry and productivity; Galloway and Cowling 1978). We ensured that collinearity would not severely influence model estimation by selecting variables between which the correlation coefficients were <0.7 (Dormann et al. 2013). Furthermore, all variables were centred and scaled for genotype–environment correlations.

Genotype–environment correlations were predicted using an LFMM analysis to identify correlation between single loci and environmental predictors and a population‐based pRDA to investigate multilocus signals of selection. While both methods rely on the simplifying assumption that allele frequencies are linearly correlated with environmental variables, they were well suited for our sampling design because they can account for the confounding effects of underlying neutral genetic structure (Forester et al. 2018; Rellstab et al. 2015). For this analysis, we focused on native populations (36 populations) to minimize biases due to the recent human‐mediated introduction of locally adapted genotypes to environments where the genetic variants may be suboptimal. For the LFMM, we used the lfmm2 function of the LEA package (Frichot and François 2015), which implements a frequentist approach using least‐squares estimates. In line with population structure analyses, we specified two as a number of latent factors. We adjusted *p*‐values for multiple testing using Benjamini–Hochberg's procedure (Benjamini and Hochberg 1995). For the pRDA, we used the native populations' allele frequency as a response and the four climatic variables as predictors to identify the relative contributions of these factors to the resulting genetic divergence. Specifically, we accounted for the influence of geographic location and population structure (demographic history) on spatial patterns of genetic variation by including geographical coordinates and admixture proportions for *K* = 2 as conditioning variables. To disentangle the effects of climate, geographic location and demographic history on the distribution of genetic variation across the native range, we conducted a pRDA‐based variance partitioning analysis by comparing the variance explained by a model including all variables (the full model: climate + geographic location + demography) to the variance explained by one set of variables (e.g., climate) when the other variables were included as conditional variables (e.g., geographic location and demography). Then, we identified outlier loci based on their extreme loading (±2.5 standard deviations) on the first axis (see https://popgen.nescent.org/2018‐03‐27_RDA_GEA.html). Finally, to visualize patterns of adaptive variation in the native range, we ran an additional RDA on the putatively adaptive candidates using the loci detected by both the LFMM and the pRDA.

### 2.6 Visualizing the Landscape of Genotype–Environment Correlations

To explore whether the genotype–environment analysis could be used to predict the success of source populations in non‐native regions, we used the loci associated with environmental variables in both the pRDA and the LFMM to extrapolate an index of genetic composition correlated with climates based on the native range (the “genotype–environment index,” sometimes called “adaptive index”; Forester et al. 2018). Specifically, we used the scores of the environmental variables along the first axis of the adaptively enriched RDA to calculate the genotype–environment index for each pixel of the landscape (Steane et al. 2014). As in Capblancq and Forester (2021), the genotype–environment index was calculated as the sum of each climatic variable score (loading) on the axis multiplied by its standardized value at each focal pixel; as such, it shows how loci correlate with environmental predictors and represent similarities on a raster. Divergence is depicted by negative and positive index values, which respectively correspond to negative and positive scores along the RDA axis. To explore the extent of genetic changes needed for the genetic composition of source populations to correlate with environmental conditions in the invaded range (i.e., the genetic offset), we computed the difference between the genotype–environment index of the environmental pixel where a tench population was introduced and that of the most closely related native population based on pairwise *Fst*. Then, we classified the lower and upper tertile as having low and high differences between the index values, while the rest fell into intermediate genotype–environment index value category. We interpret a small value as evidence that a genotype occurs in the non‐native range under similar environmental conditions as in the native range. A large value, on the other hand, indicates a mismatch between the environmental conditions under which a genotype occurs in the native range and those under which it occurs in the non‐native range.

## 3 Results

### 3.1 Genetic Data

The genomic data consisted of a total of 4.0 million sequences from which we called, initially, a total of 248,092 SNPs. After filtering for genotype depth, removing 135 individuals with high missingness rate and retaining only one SNP per locus, the final dataset comprised 7958 SNPs for 633 individuals with an overall genotype missingness rate of 6.42%. These individuals were sampled from 36 native sites, 40 introduced sites and 19 farms (Table S1).

### 3.2 Population Structure

At the broadest spatial scale, the optimal number of clusters could not be discerned due to the absence of a clear minimum in ADMIXTURE's cross‐validation errors (Figure S2). A notable bend in the cross‐validation error curve occurred after *K* = 2 (CV = 0.142), 3 (CV = 0.132) and 7 (CV = 0.123), suggesting that these values could approximate different population structures. While there was a continuous decline among nearly equal CV values (i.e., CV range for *K* = 8–20: 0.124–0.117), new clusters were generally assigned to sampled populations in small proportions. Within the native range, clustering results at the broad spatial scale revealed a longitudinal cline in allelic frequency between two clusters (Figure 1C). Populations where average individual assignment to the first cluster exceeded 75% were located in the eastern region (i.e., Ukraine, Kazakhstan, Russia, Romania, Bulgaria, Slovakia, Estonia and Turkey; hereafter Eastern cluster). Conversely, populations where individuals were predominantly assigned to the second cluster were found in the western region (i.e., France, Germany, Switzerland, Italy, Sweden, Poland, Bosnia; hereafter Western cluster). Considerable admixture was observed in the middle of the range (Figure 1A,B). Analyses at *K* = 3 and *K* = 7 revealed two distinct clusters within the native range comprising the Ghazian (Iran) and Hessen (Germany) populations (Figure S2). However, no introduced populations shared genetic composition with these two clusters, eliminating them as potential source populations. Further subdivision within the native range was indicated by: (1) a notable bent in the cross‐validation error curve at *K* = 4 (CV = 0.147) (i.e., Eastern/Western, Ghazian [Iran] and Hessen [Germany]) when examining native populations separately from introduced and farmed populations (Figure S3); and (2) the PCA (Figure 1C), which highlighted a longitudinal cline in allelic frequency along PC1 and separated Ghazian (Iran) and Volga River (Russia) populations from the rest along PC2.

Some introduced populations were predominantly assigned to the “Eastern cluster” (i.e., Australia, New Zealand, Spain and United States) or the “Western cluster” (i.e., South Africa). At the broad spatial scale, *K* = 7 separated a group of introduced populations in Turkey from other Eastern populations; however, only traces of this cluster were present in some native populations assigned to the Eastern cluster (Figure S2). When introduced populations were analysed separately from native and farmed ones, analyses inferred 12 clusters (CV = 0.059) (Figure S4). For example, tench introduced to China were genetically distinct. Those introduced to Canada and United States formed two distinct groups, and those introduced to Turkey clustered into three groups (Figure S4). At this scale, tench collected throughout Tasmania, New Zealand (excluding Hamilton) and Spain were assigned to a single cluster. Similarly, another group of introduced populations (South Africa, Bosnia and Italy) clustered together. Moreover, while most farmed populations exhibited admixed genetic composition, some were predominantly assigned to either the Eastern (i.e., Spain and Bulgaria) or Western (i.e., France, Germany and Italy) cluster. In separate analyses from native and introduced populations, farmed populations clustered into four groups (CV = 0.185) corresponding to the Western (Italian, French and German stocks) and Eastern (Spanish and Bulgarian stocks) clusters, along with two farms from Hungary and Czech Republic (Figure S5).

**FIGURE 2:**
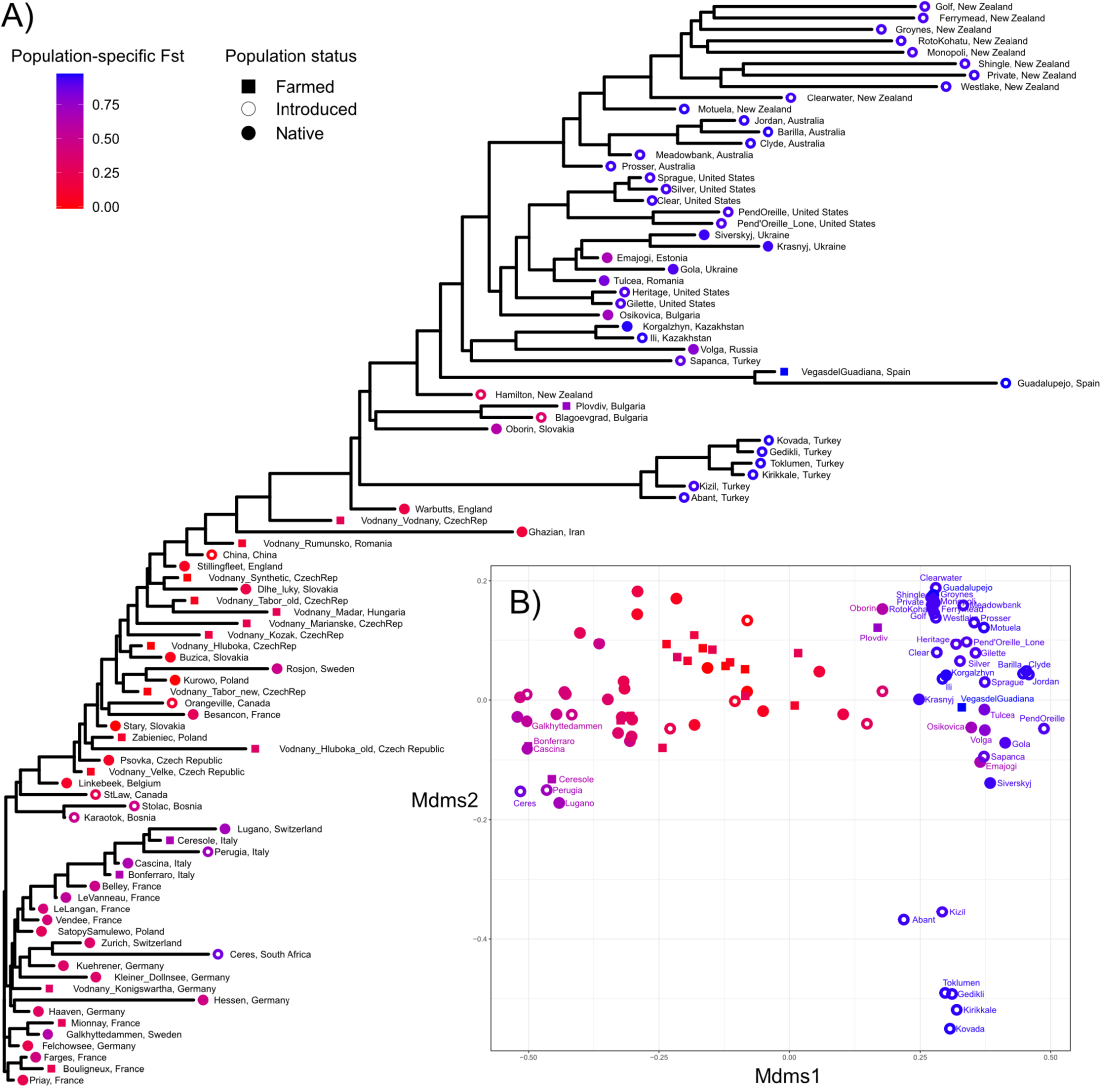
Neighbour‐joining unrooted tree (A) and multidimensional scaling (MDS) plot (B) based on pairwise *Fst* values of tench (*Tinca tinca*) populations used in this study. The colour of each population represents the magnitude of the population‐specific *Fst* value for each population, and shape indicates the status (farmed, introduced or native) of the population.

Ten of the fourteen populations with the lowest population‐specific *Fst* (<0.10) were located in Central Europe (Table S2). These included five farmed stock and one native population from Czech Republic, two native populations from Slovakia and one native and one farmed population from Poland (Figure 2). Additionally, among populations with low *Fst*, there was a population introduced to China, along with two native populations from England and Belgium, and a farmed population from Romania. In contrast, among 35 populations with the highest population‐specific *Fst* (>0.9), 30 were introduced populations; including those introduced to the United States, Australia/New Zealand, Spain and Turkey. This high‐*Fst* group included three native populations from Ukraine, a native population from Kazakhstan and a farmed population from Spain. Locations with the lowest population‐specific *Fst* were placed in the middle of the neighbour‐joining tree inferred from pairwise *Fst* values (Figure 2). On one side of the tree, native populations from Western Europe formed multiple subclusters (e.g., France, France/Germany, Switzerland/Germany and Italy/France). On the other side, native populations from the eastern part of the range (e.g., Iran, Ukraine and Turkey) and many introduced populations formed subclusters along this axis of expansion (e.g., United States and Australia). Finally, the first axis of the MDS plot characterized the divergence between Italian populations and invasive populations with high population‐specific *Fst*. Meanwhile, the second axis highlighted the differentiation between populations introduced to Turkey and those introduced to Spain and New Zealand.

### 3.3 Patterns of Genetic Diversity and Differentiation

**FIGURE 3:**
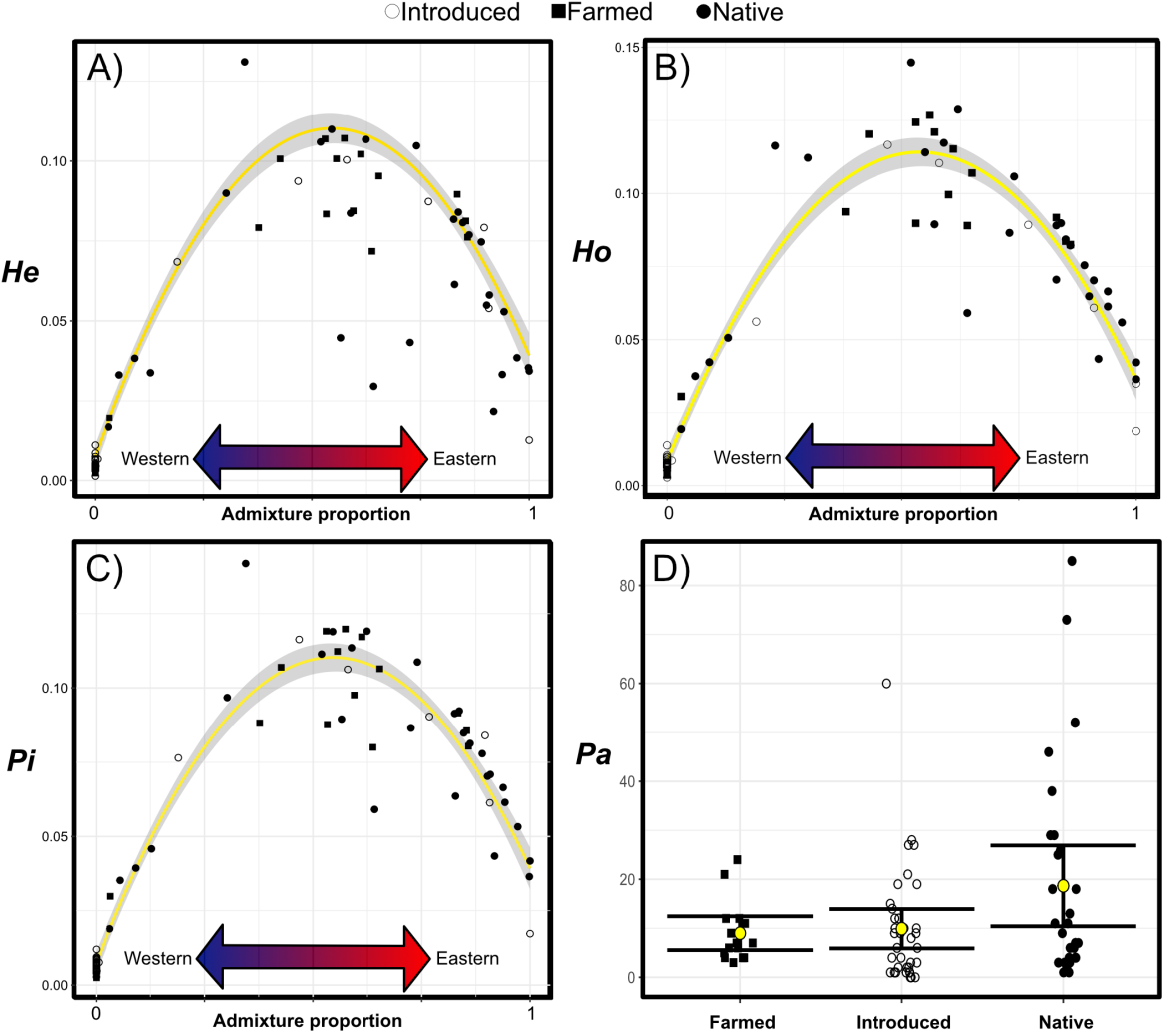
Relationship among genetic diversity metrics, admixture proportion and status (introduced, farmed and native) among tench (*Tinca tinca*) populations. Genetic diversity metrics include expected heterozygosity, *He* (A), observed heterozygosity, *Ho* (B), nucleotide diversity, *Pi* (C), and number of private alleles, *Pa* (D). Populations assigned to the Western genetic cluster were characterized by high admixture proportion and populations assigned to the Eastern genetic cluster by low values. The yellow line indicates the predicted relationship between admixture proportion and *He*, *Ho* and *Pi* with 95% confidence intervals (shaded area). The *Pa* plot shows mean (yellow dot) with 95% confidence intervals.

Variations in *He*, *Pi* and *Ho* among tench populations were primarily attributed to their ancestral genetic composition rather than their recent introduction history (Figure 3A–C and Table S2). Likelihood‐ratio tests indicated a relationship between all three metrics and populations' ancestral genetic composition (*F*(2,77) = 120.51, 272.76, 265.55, *p* < 0.001, for *He*, *Pi* and *Ho*), with no effect of population status (native, introduced and farmed) (*F*(2,77) = 0.23, 0.36, 2.58; *p* = 0.75, 0.70, 0.08 for *He*, *Pi* and *Ho*, respectively). The final models, including ancestral genetic composition as the sole predictor, explained 86%, 93% and 93% of the variance in *He*, *Pi* and *Ho*. These models predicted lower genetic variation in populations with a pure Eastern genetic composition compared to those with pure Western composition, while populations with intermediate admixture proportion showed higher levels of genetic diversity. In contrast, variation in *Pa* showed a different pattern across the range with recent introduction history playing a significant role (*F*(2,77) = −98.14, *p* < 0.001) rather than ancestral genetic composition (*F*(2,77) = −4.95, *p* = 0.08; Figure 3D). The final model, which only explained 9.8% of the variance in *Pa*, predicted higher *Pa* for native populations (mean [95% CI] = 18.9 [10.4–26.9]) compared to introduced (mean [95% CI] = 9.92 [5.90–13.9]) and farmed (mean [95% CI] = 9 [5.55–12.5]) populations.

### 3.4 Genotype–Environment Correlations

The pRDA‐based variance partitioning analysis revealed a high degree of collinearity among the explanatory variables, with much of the genetic variance (52%) explained by the full model unable to be uniquely attributed to climate, geographic location or demographic history (Table 1). Together, climate, geographic location and demographic history explained 78% of the total genetic variance across native tench populations. When controlling for geographic location and demographic history, the effect of climate was significant, explaining 6% of total genetic variation (7% of the explained variation). Geographic location accounted for 4% of total genetic variation, while demographic history alone accounted for 31% of the total genetic variance.

**TABLE 1:** The influence of climatic variables, geographic location and population structure on genetic variation decomposed with pRDA.

pRDA models	Inertia	*R* ^2^	*p*	Explainable variance (%)	Total variance (%)
Full model (clim. + geog. + stru.)	863.6	0.785	0.001	1	0.78
Climate (clim. + [geog., demo.])	61.2	0.056	0.036	0.07	0.06
Geography (geog. + [clim., demo])	43.4	0.039	0.001	0.05	0.04
Demography (demo. + [geog., clim.])	339.3	0.308	0.001	0.40	0.31
Confounded	396.4			0.52	0.41
Total unexplained	236.7				0.22
Total inertia	1100.3				1

**FIGURE 4:**
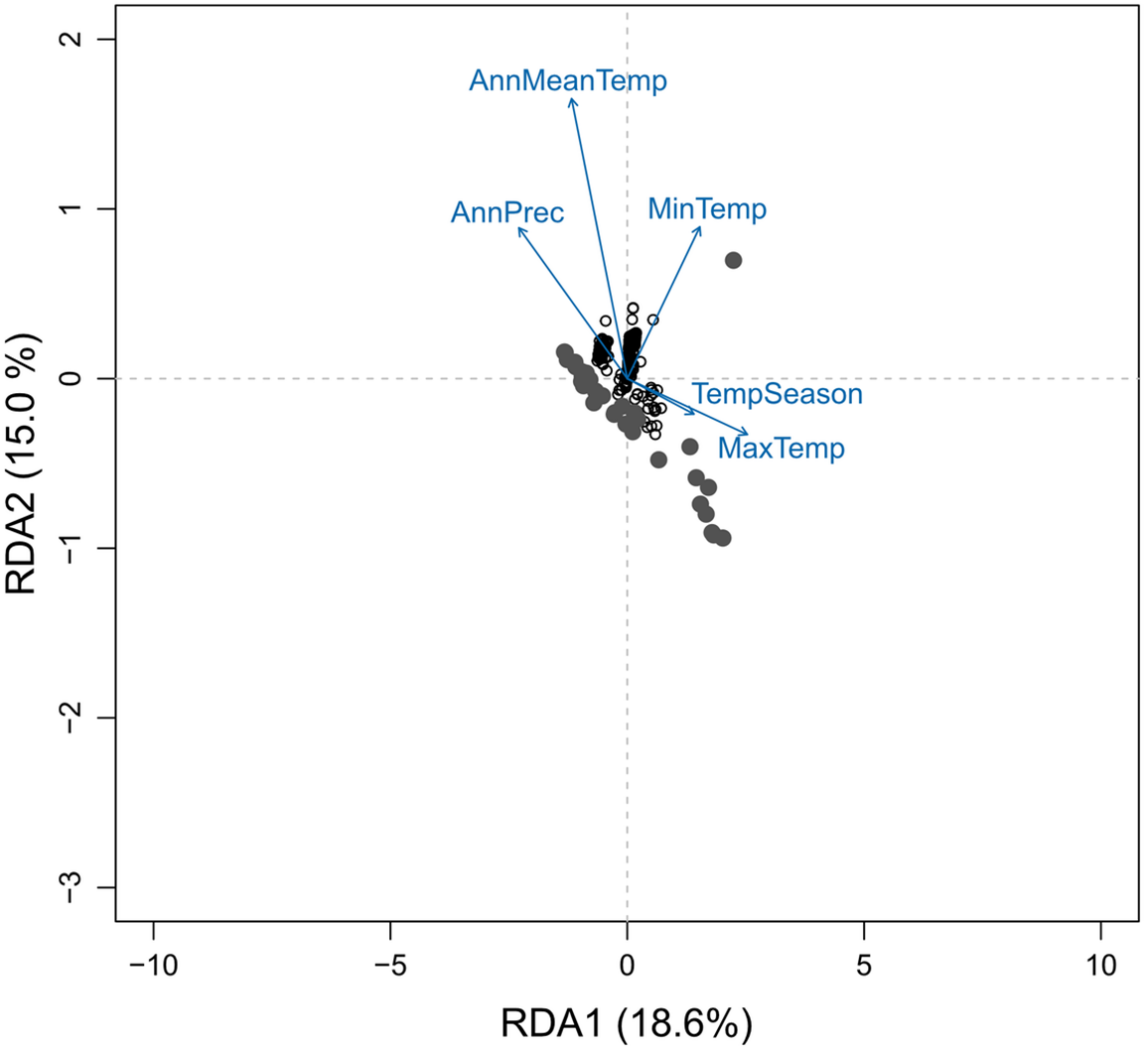
Redundancy analysis (RDA) on 642 putatively adaptive candidate loci associated with environmental variation in the native range of tench (*Tinca tinca*) populations used in this study. The plot shows the SNPs as small empty circles, environmental variables as blue arrows and native populations as large grey circles. AnnMeanTemp, annual mean temperature; AnnPrec, annual precipitation; MaxTemp, maximum summer temperature; MinTemp, minimum winter temperature; TempSeason, annual temperature range.

The LFMM and the pRDA identified 357 and 408 candidate markers, respectively, with 122 shared between the two methods (643 loci combined). In the pRDA, many candidate markers were most strongly associated with annual mean temperature (237 loci), followed by minimum temperature of the coldest month (102 loci), maximum temperature of the warmest month (45 loci), annual precipitation (22 loci) and temperature seasonality (2 loci). Subsequent RDA on the adaptive loci detected by the LFMM and RDA explained 36.4% of the total variance (Figure 4). When examining the projection of individual populations in the (putatively) adaptively informed RDA space, populations of similar ancestry (e.g., Eastern cluster and Western cluster) tended to be close to each other. Populations assigned to the Western cluster were generally associated with greater annual precipitation, warmer winter temperatures, colder summer temperatures and smaller annual temperature ranges than Eastern populations (Figure 4).

### 3.5 Genotype–Environment Index

**FIGURE 5:**
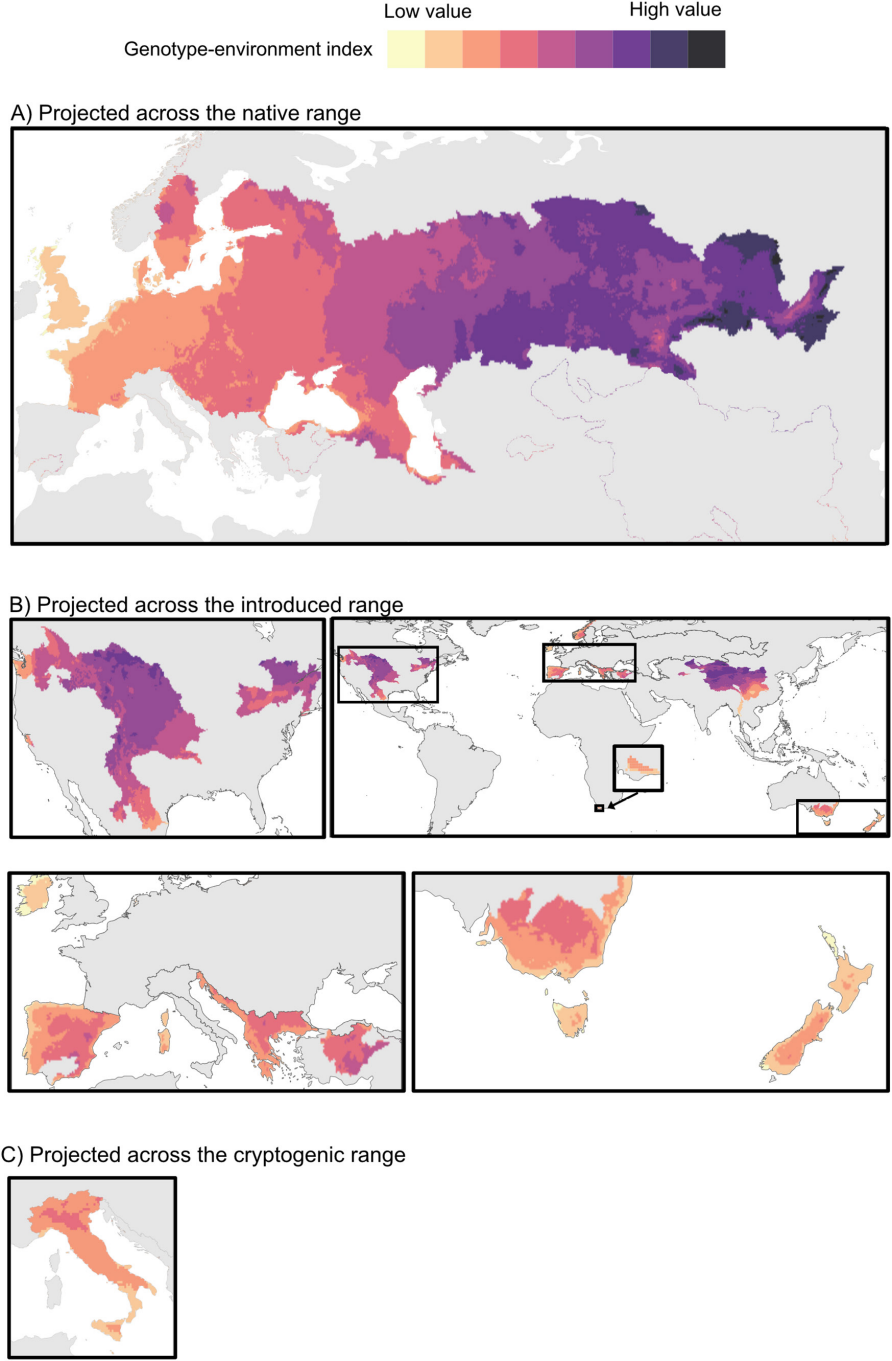
Genotype–environment index for tench (*Tinca tinca*) extrapolated across (A) the native range, (B) the introduced range and (C) the cryptogenic range (i.e., of uncertain origin). The index estimates similarities (similar colours) and differences (different colours) in genetic variation as a function of environmental predictors. It is standardized to an unshown scale and directly comparable between the maps.

In the native range, extrapolations on RDA1 showed a gradient contrasting Western (low value) and Eastern (high value) regions (Figure 5A and Table 2). The absolute value of this genotype–environment index allows grouping of populations based on similar index values. For example, populations located in Western Europe (United Kingdom, France, Belgium and the Netherlands) were characterized by high values, while those in Central Europe, Southeastern Europe and Transcaucasia (Georgia and Azerbaijan) by mid‐high values. In contrast, the western parts of Eastern Europe (Belarus and Ukraine) were characterized by mid to mid‐low values, transitioning into low values east of the Caspian Sea. Some freshwater ecoregions where tench was introduced exhibited high genotype–environment index values (e.g., Central and Eastern North America and China) while others were characterized by low values (e.g., Australia, New Zealand, Spain and Italy) (Figure 5B,C).

**TABLE 2:** Genetic offset for introduced populations of tench.

Introduced population	Source population	Offset	Relative difference
Toklumen, Turkey	Emajogi, Estonia	0.47	Low
Clearwater, New Zealand	Warbutts, England	0.70	Low
Ferrymead, New Zealand	Warbutts, England	0.70	Low
Golf, New Zealand	Warbutts, England	0.70	Low
Groynes, New Zealand	Warbutts, England	0.70	Low
Monopoli, New Zealand	Warbutts, England	0.70	Low
Private, New Zealand	Warbutts, England	0.70	Low
RotoKohatu, New Zealand	Warbutts, England	0.70	Low
Shingle, New Zealand	Warbutts, England	0.70	Low
Westlake, New Zealand	Warbutts, England	0.70	Low
Kizil, Turkey	Emajogi, Estonia	0.70	Low
Stolac, Bosnia	Zurich, Switzerland	0.74	Low
Kirikkale, Turkey	Emajogi, Estonia	0.88	Low
Ceres, South Africa	Zurich, Switzerland	0.93	Low
Perugia, Italy	Cascina, Italy	0.93	Low
Blagoevgrad, Bulgaria	Krasnyj, Ukraine	1.00	Low
Gedikli, Turkey	Emajogi, Estonia	1.02	Low
Abant, Turkey	Emajogi, Estonia	1.25	Low
Sprague, United States	Emajogi, Estonia	1.53	Low
PendOreille, United States	Emajogi, Estonia	1.69	Low
Kovada, Turkey	Emajogi, Estonia	1.69	Low
Silver, United States	Emajogi, Estonia	2.14	Low
Karaotok, Bosnia	Linkebeek, Belgium	2.24	Low
Heritage, United States	Emajogi, Estonia	2.29	Low
Clear, United States	Emajogi, Estonia	2.42	Low
Pend'Oreille_Lone, United States	Emajogi, Estonia	2.49	Low
Gilette, United States	Emajogi, Estonia	2.72	Low
Ili, Kazakhstan	Korgalzhyn, Kazakhstan	2.90	Low
Clyde, Australia	Emajogi, Estonia	3.29	Medium
Sapanca, Turkey	Emajogi, Estonia	3.62	Medium
Orangeville, Canada	Stary, Slovakia	4.01	Medium
Meadowbank, Australia	Emajogi, Estonia	4.15	Medium
Guadalupejo, Spain	Warbutts, England	4.44	Medium
China, China	Kurowo, Poland	4.51	Medium
Jordan, Australia	Emajogi, Estonia	4.65	Medium
Barilla, Australia	Emajogi, Estonia	5.04	Medium
Motuela, New Zealand	Emajogi, Estonia	5.13	High
Prosser, Australia	Emajogi, Estonia	5.72	High
Hamilton, New Zealand	Krasnyj, Ukraine	7.40	High
StLawrence, Canada	Linkebeek, Begium	8.49	High

*Note:* The genetic offset is defined as the difference between the genotype–environment index of the introduced populations and that of the most closely related native population (i.e., the source population) based on pairwise *Fst*.

Of the 40 introduced populations, 27 exhibited relatively small differences (<2.83) in their genotype–environment index compared to their most genetically similar native populations (Table 2). All of the tench populations introduced to Australia and Canada exhibited intermediate‐to‐large genetic offset values. Other populations with intermediate‐to‐large values included two populations introduced to New Zealand and single populations introduced to Turkey, Spain and China.

## 4 Discussion

Although rapid evolution is hypothesized to play an important role in facilitating invasiveness, the relative importance of long‐term evolutionary processes in shaping population structure and genetic diversity within native source populations on invasiveness remains understudied. We show that the distribution of genetic diversity across native, introduced and farmed populations of tench has been shaped by both historical and contemporary processes. Specifically, genetic diversity levels differed between two geographically distinct genetic clusters corresponding to the previously described Eastern and Western phylogroups (Lajbner et al. 2010; Lajbner, Linhart, and Kotlík 2011; Lajbner and Kotlik 2011) with a peak in genetic diversity coinciding with admixture at the core of the native range. When accounting for this variation, introduced populations exhibited levels of genetic variation similar to those of their native source populations. Finally, populations that have successfully established and persisted in non‐native regions originated from native source populations with varying levels of genetic variation (e.g., admixed regions and Eastern and Western clusters) and their source genotypes did not always match environmental conditions in the introduced range.

### 4.1 Population Structure and Evolutionary History

As expected from previous studies suggesting that tench experienced extensive admixture between Eastern and Western phylogroups corresponding to distinct glacial refugia (Karaiskou et al. 2020; Lajbner et al. 2010; Lajbner, Linhart, and Kotlík 2011; Lajbner and Kotlik 2011), tench diversity at a broad geographic scale consisted of a cline of variation in allelic frequency between two clusters. In many populations across the range, individuals had partial membership to multiple clusters with similar membership coefficients. Here, we demonstrate that, among native populations, this history of admixture resulted in a peak of genomic variation (i.e., *He*, *Ho* and *Pi*) in Central Europe, away from the known glacial refugia—a pattern documented in several other species in Europe (Petit et al. 2003; Havrdová et al. 2015; Horníková et al. 2021). In conjunction with high heterozygosity and nucleotide diversity, many of the native populations exhibited relatively low values of population‐specific *Fst* and pairwise *Fst*, a possible result of admixture which could explain the lack of clear‐cut boundaries between subpopulations across the native range at the level of analysis where population structure was examined in this paper. Interestingly, a previous study of marine fishes demonstrated that species with low pairwise *Fst* in their native range were more likely to be successful invaders (Gaither, Bowen, and Toonen 2013). Our findings suggest that low pairwise *Fst* might similarly be applied in predicting invasiveness in freshwater systems, as it may indicate species with a successful establishment and persistence throughout their long‐term demographic and colonization history in the native range.

While uneven sample sizes between populations hamper conclusions related to the exact number of distinct populations, tench diversity was also characterized by hierarchical substructuring particularly apparent within introduced populations. For example, at finer admixture resolution, at least five clusters were apparent among introduced populations. Additionally, these introduced populations also exhibited high population‐specific *Fst* values, highlighting their distinctiveness relative to the broader pool of genetic diversity in tench populations. This distinctiveness may be attributed to genetic drift and adaptive changes exacerbated by limited gene flow. In addition, despite exhibiting relatively low population‐specific *Fst* values, some native populations were characterized by high numbers of private alleles, another metric of genetic uniqueness. For example, the Osikovica (Bulgaria) and Ghazian (Iran) populations exhibited private allele counts that were three‐ to fourfold greater than the average count across native populations. Our results indicate that limited gene flow combined with genetic drift and/or selection occurring at or near loci with private alleles (Slatkin 1985) has contributed to fine‐scale patterns of genetic divergence across the range.

### 4.2 Patterns of Genetic Variation Between and Within Introduced, Native and Farmed Populations

When accounting for the influence of long‐term evolutionary processes on population structure across the range, we found that introduced populations did not exhibit lower genetic diversity (*He*, *Ho* and *Pi*) compared to native source populations. Theoretically, if the founder population includes ≥10 individuals, it is predicted that 95% of the heterozygosity contained in a source population at polymorphic loci with minor allele frequency > 0.05 should be retained (Kimura and Ohta 1969). Therefore, the retention of genetic variation in introduced tench observed here might stem from relatively high propagule pressure in the founder populations. Alternatively, for the tench population in the St. Lawrence River, rapid population expansion following the introduction of approximately 36 founder individuals has been proposed as a likely contributor to the retention of genetic variation (Bernos et al. 2023). However, introduced populations exhibited significantly fewer private alleles than native populations. The number of private alleles is particularly susceptible to reductions in population size (Nei, Maruyama, and Chakraborty 1975) and introduced populations have had less time for de novo mutations to arise compared to native populations. Collectively, our results challenge the paradoxical aspect of invasion genetics by demonstrating that some introduced populations do not have lower genetic variation than their native source populations and may therefore retain their fitness and evolutionary potential in new habitats (Dlugosch et al. 2015; Dlugosch and Parker 2008; Edelaar et al. 2015; Jaspers et al. 2021; Uller and Leimu 2011).

Importantly, the impact of long‐term processes on genetic variation, such as population differentiation during the last glaciation, prevailed over the effects of recent founder effects following introductions to non‐native regions. This was evident in the variation in genetic diversity observed between and within invaded and native regions. Specifically, we found that: (1) native, introduced and farmed populations assigned to the Eastern cluster were characterized by lower heterozygosity and nucleotide diversity than the Western cluster; and (2) populations with substantial contributions from both Eastern and the Western phylogroups exhibited higher levels of heterozygosity and nucleotide diversity than pure populations. Yet, despite a 10‐fold variation in genetic diversity among Eastern, Western and admixed native source populations, all of them successfully contributed to the establishment of introduced populations. These results imply that low total genetic diversity alone, per se, did not necessarily prevent successful invasions.

### 4.3 Role of Prior Adaptation in Promoting Invasion Success

While patterns of local adaptation existed in the native range, as estimated by genetic–environment correlations in the native range, these did not serve as prior adaptations crucial to the success of the introductions to non‐native environments. In particular, some introduced populations of tench became widespread invaders (e.g., in the St Lawrence River in Canada and Tasmanian waters) despite facing potential adaptive challenges in the introduced range as shown by mismatches between source genotypes and environmental conditions in the introduced range. The invasive population of the St Lawrence River, characterized by admixed ancestry, showed high levels of genetic variation in the introduced range. In contrast, the success of the Tasmanian population is more paradoxical; this introduced population exhibits low genetic variation—the consequence of bottleneck events in native source populations rather than the introduction process—and simultaneously faces potential adaptive challenges. The evidence of invasion success despite adaptive mismatches warrants caution regarding the interpretation of climate‐matching approaches (e.g., Bomford, Barry, and Lawrence 2010; Hubbard, Drake, and Mandrak 2023) that rely on prior adaptation hypothesis for predicting the relative success of introduced populations, especially for species with broad native ranges.

It is worth considering that certain limitations inherent to the methodological approaches adopted in our study may have influenced the strength of our inference based on correlations between genotypes and the environment. First, due to the use of GBS, many adaptive genes may not have been represented in our dataset. Second, the lack of genetic resources—such as an annotated reference genome for tench—hampers conclusions related to the significance of potentially adaptive variants (Funk et al. 2019), a desirable topic for future investigations. Third, adaptation to aspects of the environment shared between the native and introduced range that was not considered in our analysis (e.g., water chemistry, local ecological community and human‐altered habitats) could facilitate invasiveness as genetic–environment indexes are heavily influenced by the choice of predictor variables (Lachmuth et al. 2023). Fourth, limited sample sizes (≤25 individuals per population) may have reduced the precision of our estimates of allele frequencies, expected heterozygosity and the number of private alleles. Future studies integrating whole‐genome resequencing with field experiments and incorporating morphological, physiological and/or ecological data will provide deeper insights into the mechanisms facilitating invasiveness. Despite these limitations, it is noteworthy that populations with very similar genetic backgrounds were able to establish viable populations and persist in environments characterized by strikingly different climatic and ecological conditions.

## 5 Conclusions

Our study showed that the current distribution of genetic variation in a broadly introduced fish is primarily shaped by long‐term evolutionary processes rather than recent introductions during the Anthropocene. Historical genetic links to two distinct ancestral populations and subsequent admixture have played a crucial role in shaping genetic diversity across invasive, native and farmed populations. Despite the variations in genetic diversity among native source populations, introduced populations managed to establish and thrive in distant locations. Surprisingly, adaptation to native environmental conditions may not always contribute to the invasion success of tench populations, as shown by genotype–environment mismatches and the successful establishment of genetically similar populations in diverse non‐native environments. Finally, some introduced populations became invasive despite harbouring low levels of genetic variation and lacking prior adaptation to the invasive range. These results challenge the general applicability of the invasive species paradox and highlight the complex interplay between historical processes and contemporary introductions in shaping genetic diversity and ecological outcomes in introduced populations.

These results also suggest that factors beyond genetic adaptation to specific environments may play an important role in the success of introduced or invasive populations in new habitats. For example, epigenetic variation—mechanisms promoting phenotypic variation without changing the underlying DNA sequence (Richards, Verhoeven, and Bossdorf 2012)—could facilitate the establishment of populations that were not adapted to their new environment and/or exhibited reduced genetic variation prior to the introduction. It has been hypothesized that species, like tench, capable of colonizing new areas and expanding their native range might better regulate gene expression via epigenetic mechanisms (Marin et al. 2020; Schrey et al. 2012). It is also plausible that other factors, which include physiological plasticity, reproductive strategies or interactions with other species, could contribute to ecological success in new environments. Understanding these mechanisms is crucial for the effective management of introduced populations and the conservation of biodiversity in rapidly changing environments.

## Conflicts of Interest

The authors declare no conflicts of interest.

## Benefit Sharing Statement

A research collaboration was developed between all collaborators, including scientists in academic and government agencies in Canada, Czech Republic, Japan and Australia. The research addresses an important topic for conservation, namely the evolutionary mechanisms facilitating successful invasions, using tench, an invasive species with adverse consequences for native biodiversity and ecosystems, as a model.

## Supporting information


Data S1:

## Data Availability

R Code along with associated data files (i.e., final filtered SNP file in VCF format, geographic location and environmental data) will be uploaded on GitHub.
