# Supplementary material for: Assessing the Impacts of Adaptation to Native‐Range Habitats and Contemporary Founder Effects on Genetic Diversity in an Invasive Fish
Source: Evol Appl. 2024 Oct 4;17(10):e70006. doi: 10.1111/eva.70006 (PMC11450252; doi:10.1111/eva.70006)
Supplement: Supplementary file 1 — Data S1: [file EVA-17-e70006-s001.docx]

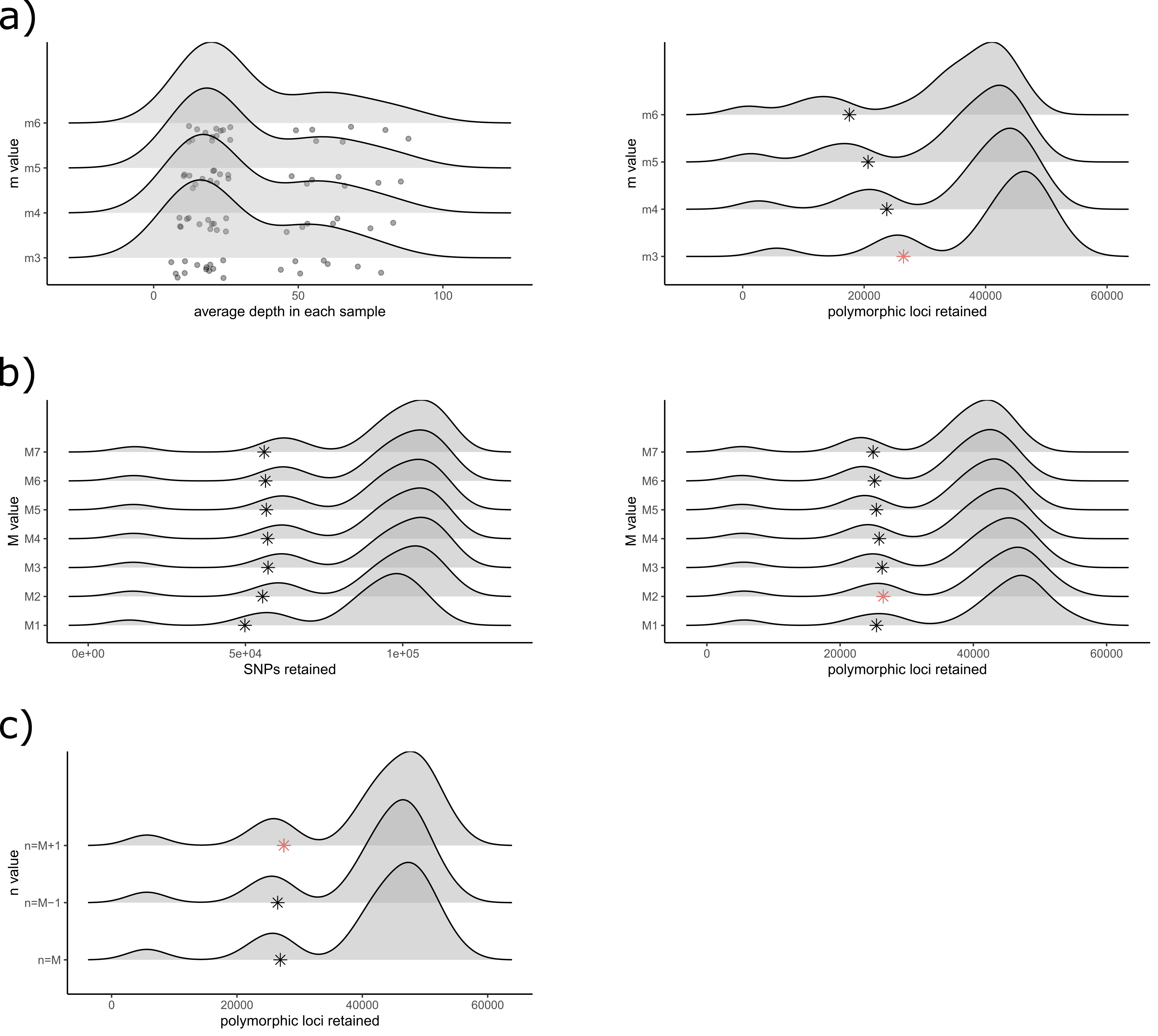


Figure S1: Effects of varying a) m, b) M, and c) n on average depth, number of SNPs retained and number of polymorphic loci retained based on the Tench (*Tinca tinca*) samples used in this study.


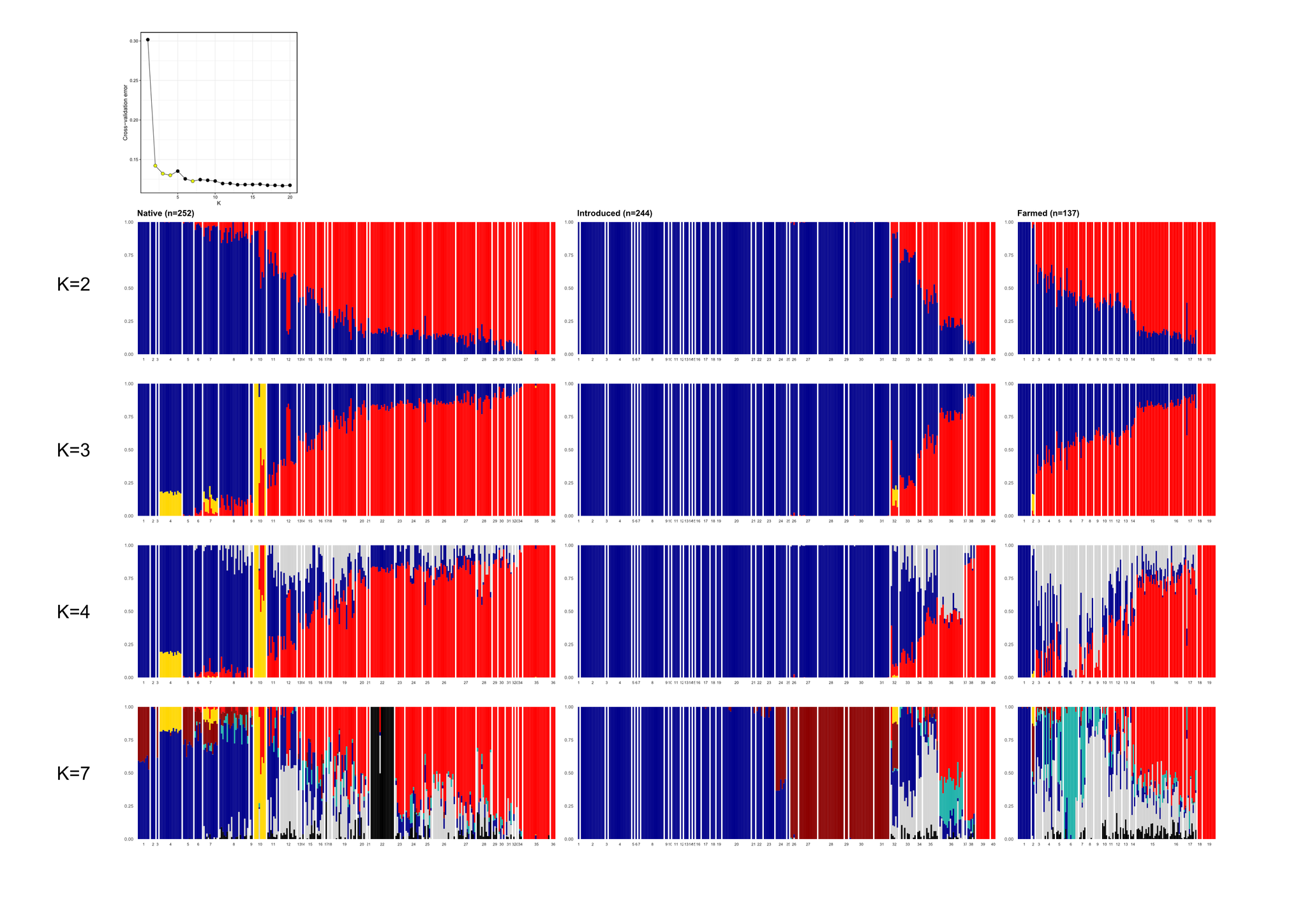


Figure S2: Cross-validation and admixture plots inferred for a range of potential K for Tench (*Tinca tinca*) at the broad spatial scale. The numbers correspond to sampling locations listed in Table S1.


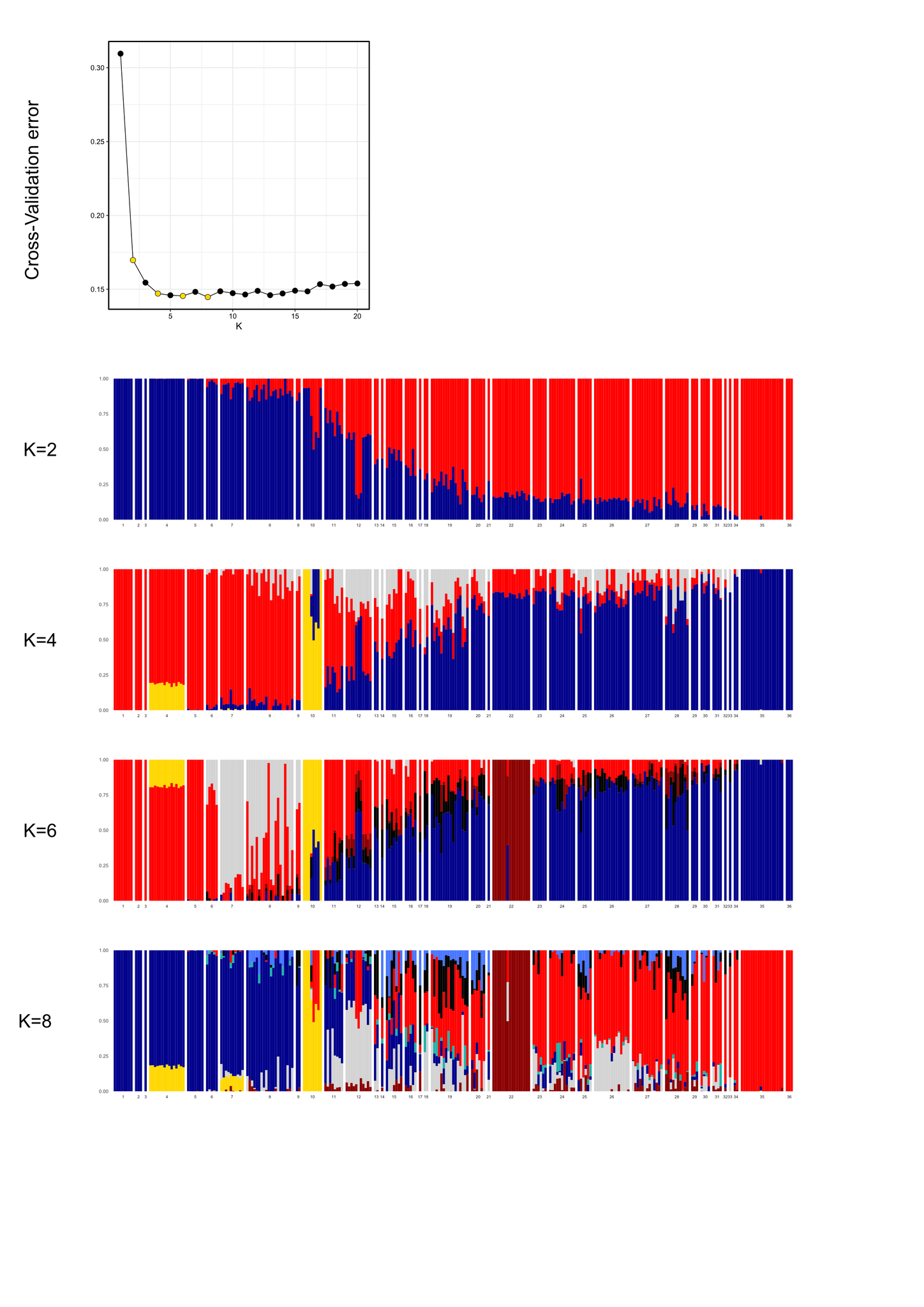


Figure S3: Cross-validation and admixture plots inferred for a range of potential K for Tench (*Tinca tinca*) sampled across native populations (n=253). The numbers correspond to sampling locations listed in Table S1.


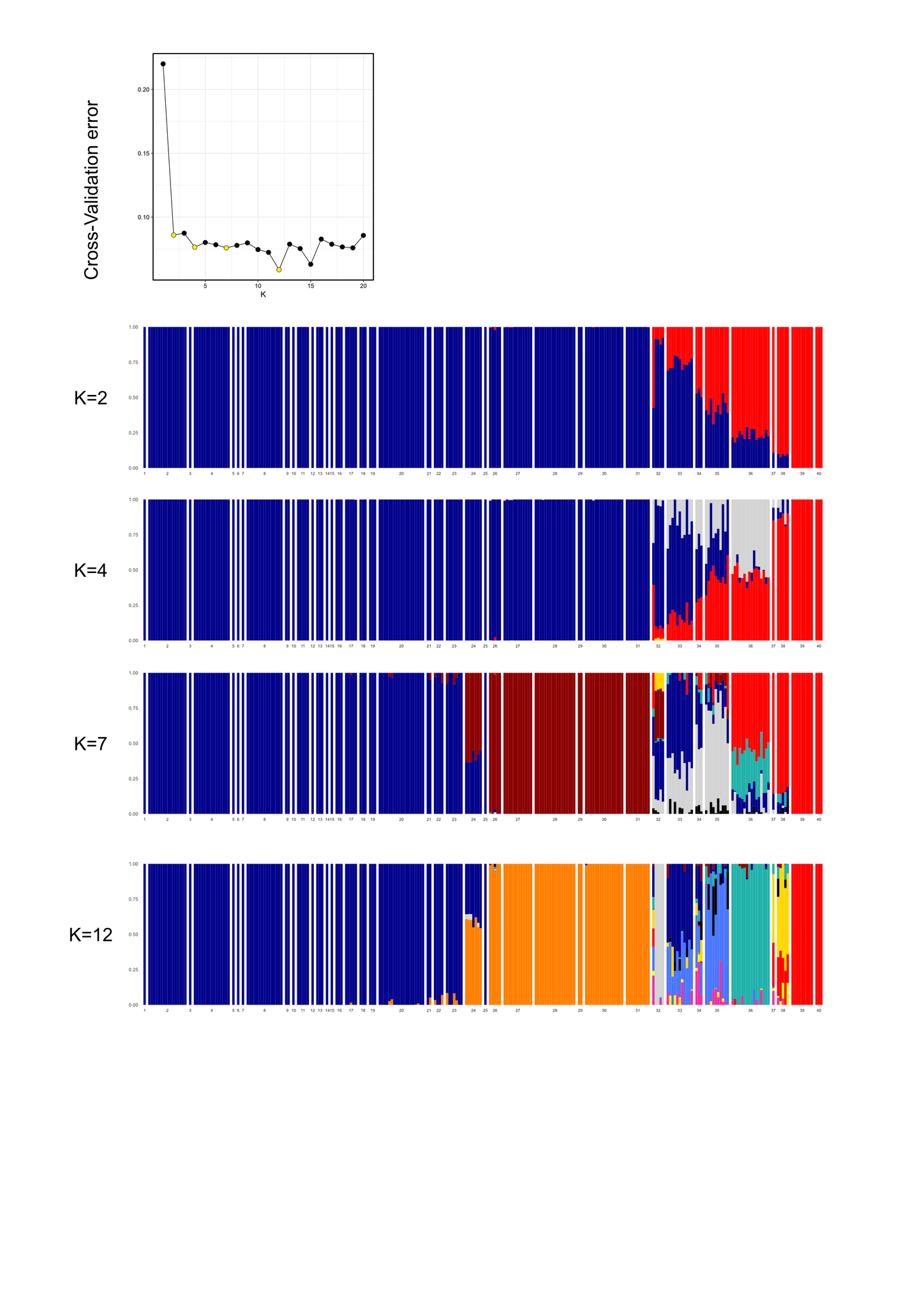
Figure S4: Cross-validation and admixture plots inferred for a range of potential K for Tench (*Tinca tinca*) sampled across introduced populations (n=244). The numbers correspond to sampling locations listed in Table S1.


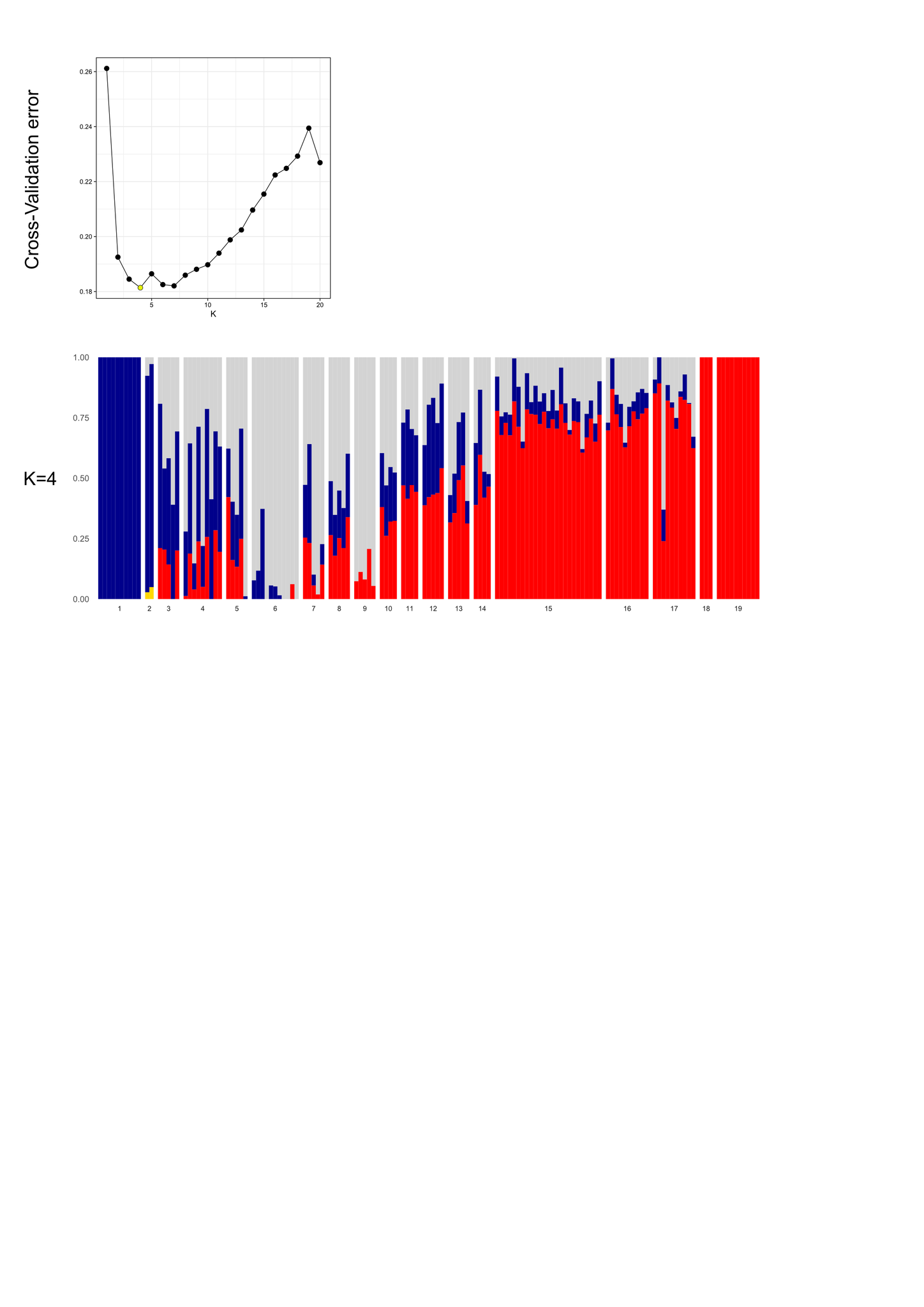


Figure S5: Cross-validation and admixture plots inferred for a range of potential K for Tench (*Tinca tinca*) sampled across farmed populations (n=137). The numbers correspond to sampling locations listed in Table S1.

## Supplementary materials

Table S1: Sampling locations for native, introduced, and farmed Tench (*Tinca tinca*) populations used in this study, sample size (N), and admixture proportion (Q) for K=2. Index corresponds to the population labels appearing in Fig. S2, S3, S4, and S5.

| **Population** | **Status** | **Latitude** | **Longitude** | **Q** | **Index** |
| --- | --- | --- | --- | --- | --- |
| Vegas del Guadiana | Farmed | 38.88 | -6.88 | 0.00 | 1 |
| Plovdiv | Farmed | 42.15 | 24.72 | 0.03 | 2 |
| Vodnany Vodnany | Farmed | 49.15 | 14.18 | 0.38 | 3 |
| Vodnany Rumunsko | Farmed | 45.94 | 24.97 | 0.43 | 4 |
| Vodnany Synthetic | Farmed | 49.16 | 14.19 | 0.53 | 5 |
| Vodnany Madar | Farmed | 47.17 | 19.5 | 0.53 | 6 |
| Vodnany Tabor old | Farmed | 49.4 | 14.69 | 0.56 | 7 |
| Vodnany Marianske | Farmed | 49.97 | 12.7 | 0.56 | 8 |
| Vodnany Hluboka | Farmed | 49.05 | 14.43 | 0.57 | 12 |
| Vodnany Kozak | Farmed | 49.16 | 14.19 | 0.60 | 10 |
| Vodnany Tabor new | Farmed | 49.45 | 14.36 | 0.61 | 11 |
| Vodnany Hluboka old | Farmed | 49.05 | 14.43 | 0.64 | 9 |
| Zabieniec | Farmed | 52.05 | 21.033 | 0.65 | 13 |
| Vodnany Velke | Farmed | 49.35 | 16.02 | 0.68 | 14 |
| Bouligneux | Farmed | 46.024 | 4.993 | 0.83 | 15 |
| Mionnay | Farmed | 45.895 | 4.916 | 0.85 | 16 |
| Vodnany Konigswartha | Farmed | 51.31 | 14.33 | 0.86 | 17 |
| Bonferraro | Farmed | 45.2 | 11 | 1.00 | 18 |
| Ceresole d'Alba | Farmed | 44.8 | 7.817 | 1.00 | 19 |
| Guadalupejo | Introduced | 39.45 | -5.31 | 0.00 | 1 |
| Barilla | Introduced | - 42.825 | 147.464 | 0.00 | 2 |
| Clearwater | Introduced | -43.514 | 172.62 | 0.00 | 3 |
| Clyde | Introduced | -42.390 | 146.997 | 0.00 | 4 |
| Ferrymead | Introduced | -43.514 | 172.62 | 0.00 | 5 |
| Golf | Introduced | -43.514 | 172.62 | 0.00 | 6 |
| Groynes | Introduced | -43.514 | 172.62 | 0.00 | 7 |
| Jordan | Introduced | -42.546 | 147.085 | 0.00 | 8 |
| Meadowbank | Introduced | -42.538 | 146.733 | 0.00 | 9 |
| Monopoli | Introduced | -43.514 | 172.62 | 0.00 | 10 |
| Motuela | Introduced | -41.180 | 172.896 | 0.00 | 11 |
| Private | Introduced | -43.514 | 172.62 | 0.00 | 12 |
| Prosser | Introduced | -42.605 | 147.714 | 0.00 | 13 |
| Roto Kohatu | Introduced | -43.514 | 172.62 | 0.00 | 14 |
| Shingle | Introduced | -43.514 | 172.62 | 0.00 | 15 |
| Ili | Introduced | 45.583 | 74.445 | 0.00 | 16 |
| Gilette | Introduced | 48.379 | -117.282 | 0.00 | 17 |
| Heritage | Introduced | 48.613 | -117.362 | 0.00 | 18 |
| Pend'Oreille Lone | Introduced | 48.237 | -117.158 | 0.00 | 19 |
| PendOreille | Introduced | 48.973295 | -117.352 | 0.00 | 20 |
| Clear | Introduced | 48.742447 | -117.414 | 0.00 | 21 |
| Silver | Introduced | 48.635017 | -117.524 | 0.00 | 22 |
| Sprague | Introduced | 47.54731 | -117.653 | 0.00 | 23 |
| Sapanca | Introduced | 40.69 | 30.26 | 0.00 | 24 |
| Westlake | Introduced | -43.514 | 172.62 | 0.00 | 25 |
| Kiriniti | Introduced | 37.652 | 30.865 | 0.00 | 28 |
| Abant | Introduced | 40.603 | 31.282 | 0.00 | 29 |
| Kirikkale | Introduced | 39.694 | 33.46 | 0.00 | 30 |
| Toklumen | Introduced | 39.132 | 33.71 | 0.00 | 31 |
| Gedikli | Introduced | 37.908 | 31.333 | 0.00 | 27 |
| Kizil | Introduced | 39.574 | 33.433 | 0.01 | 26 |
| Blagoevgrad | Introduced | 42.02 | 23.089 | 0.19 | 32 |
| Hamilton | Introduced | -37.794 | 175.275 | 0.26 | 33 |
| Lijiang | Introduced | 35.815 | 103.198 | 0.47 | 34 |
| Orangeville | Introduced | 43.92 | -80.09 | 0.58 | 35 |
| StLaw | Introduced | 44.96 | -73.33 | 0.77 | 36 |
| Karaotok Park Prirode | Introduced | 43.05 | 17.8 | 0.89 | 37 |
| Stolac | Introduced | 43.08 | 17.96 | 0.91 | 38 |
| Perugia | Introduced | 43.14 | 12.197 | 1.00 | 39 |
| Ceres | Introduced | -33.3958 | 19.2893 | 1.00 | 40 |
| Savincy | Native | 49.38 | 37.02 | 0.00 | 1 |
| Korgalzhyn | Native | 50.59 | 70.29 | 0.00 | 2 |
| Senkove | Native | 49.51 | 37.69 | 0.00 | 3 |
| Volga | Native | 46.41 | 48 | 0.00 | 4 |
| Gola Pristan | Native | 46.31 | 32.31 | 0.00 | 5 |
| Tulcea | Native | 45 | 29 | 0.03 | 6 |
| Osikovica | Native | 42.94 | 24 | 0.06 | 7 |
| Emajogi | Native | 58.83 | 27 | 0.09 | 8 |
| Oborin | Native | 48.54 | 21.9 | 0.13 | 9 |
| Ghazian | Native | 37.465 | 49.33 | 0.23 | 10 |
| Warbutts | Native | 54.05 | -1.017 | 0.30 | 11 |
| Stillingfleet | Native | 53.867 | -1.083 | 0.52 | 12 |
| Kurowo | Native | 53.12 | 22.801 | 0.55 | 15 |
| Dlhe luky | Native | 48.59 | 17 | 0.57 | 14 |
| Buzica | Native | 48.55 | 21.08 | 0.59 | 13 |
| Stary | Native | 47.765 | 17.727 | 0.62 | 16 |
| Rosjon | Native | 59.165556 | 17.924 | 0.64 | 17 |
| Psovka | Native | 50.436 | 14.581 | 0.69 | 18 |
| Besancon | Native | 47.269 | 6.097 | 0.73 | 21 |
| Linkebeek | Native | 50.767 | 4.333 | 0.74 | 19 |
| Priay | Native | 46 | 5.271 | 0.83 | 20 |
| Hessen | Native | 49.92 | 8.321 | 0.83 | 22 |
| Felchowsee | Native | 53.055 | 14.131 | 0.84 | 25 |
| Satopy-Samulewo | Native | 54.07 | 21.061 | 0.85 | 24 |
| Kleiner Dollnsee | Native | 52.997 | 13.598 | 0.85 | 23 |
| Kuehrener | Native | 54.7 | 18.28 | 0.86 | 26 |
| Haaven | Native | 53.09 | 8.211 | 0.86 | 28 |
| Zurich | Native | 47.3 | 8.621 | 0.89 | 27 |
| Vendee | Native | 46.353 | -1.04 | 0.90 | 31 |
| LeLangan | Native | 46.428 | -0.958 | 0.91 | 29 |
| Galkhyttedammen | Native | 58.691111 | 16.8175 | 0.92 | 32 |
| Farges | Native | 46.50639 | 4.929167 | 0.94 | 33 |
| Belley | Native | 45.78 | 5.811 | 0.94 | 30 |
| LeVanneau | Native | 46.317 | -0.653 | 0.97 | 34 |
| Lugano | Native | 45.983 | 8.967 | 1.00 | 35 |
| Cascina Belgiardino | Native | 45.28 | 9.481 | 1.00 | 36 |

Table S2: Genetic diversity for introduced, native, and farmed populations of Tench (*Tinca tinca*). *Pa* = Number of alleles; *Ho* = observed heterozygosity; *He* = expected heterozygosity; *Pi* = nucleotide diversity; and, population-specific *Fst*

| **Population** | **Country** | **Status** | **N** | **Pa** | **Ho** | **He** | **Pi** | **Fst** |
| --- | --- | --- | --- | --- | --- | --- | --- | --- |
| Clearwater | New Zealand | Introduced | 1 | 0 | 0.007 | 0.004 | 0.007 | 0.939 |
| Groynes | New Zealand | Introduced | 1 | 0 | 0.007 | 0.003 | 0.007 | 0.941 |
| Guadalupejo | Spain | Introduced | 1 | 0 | 0.003 | 0.001 | 0.003 | 0.977 |
| Buzica | Slovakia | Native | 2 | 1 | 0.117 | 0.084 | 0.113 | 0.041 |
| Psovka | Czech Republic | Native | 2 | 1 | 0.104 | 0.082 | 0.110 | 0.071 |
| Farges | France | Native | 1 | 1 | 0.066 | 0.033 | 0.066 | 0.442 |
| Karaotok | Bosnia | Introduced | 1 | 1 | 0.060 | 0.030 | 0.060 | 0.496 |
| Ferrymead | New Zealand | Introduced | 1 | 1 | 0.008 | 0.004 | 0.008 | 0.933 |
| RotoKohatu | New Zealand | Introduced | 1 | 1 | 0.007 | 0.004 | 0.007 | 0.937 |
| Private | New Zealand | Introduced | 1 | 1 | 0.007 | 0.003 | 0.007 | 0.945 |
| Westlake | New Zealand | Introduced | 1 | 1 | 0.006 | 0.003 | 0.006 | 0.946 |
| Abant | Turkey | Introduced | 2 | 1 | 0.007 | 0.004 | 0.006 | 0.951 |
| Motuela | New Zealand | Introduced | 5 | 1 | 0.007 | 0.004 | 0.005 | 0.961 |
| LeVanneau | France | Native | 2 | 2 | 0.056 | 0.038 | 0.053 | 0.548 |
| Golf | New Zealand | Introduced | 1 | 2 | 0.009 | 0.004 | 0.009 | 0.924 |
| Monopoli | New Zealand | Introduced | 1 | 2 | 0.008 | 0.004 | 0.008 | 0.929 |
| Clear | United States | Introduced | 2 | 2 | 0.007 | 0.004 | 0.007 | 0.943 |
| Krasnyj | Ukraine | Native | 1 | 2 | 0.006 | 0.003 | 0.006 | 0.948 |
| Vodnany_Synthetic | Czech Republic | Farmed | 5 | 3 | 0.124 | 0.107 | 0.119 | -0.007 |
| Stary | Slovakia | Native | 5 | 3 | 0.129 | 0.107 | 0.119 | -0.007 |
| Vodnany_Marianske | Czech Republic | Farmed | 5 | 3 | 0.090 | 0.082 | 0.092 | 0.224 |
| Priay | France | Native | 6 | 3 | 0.089 | 0.082 | 0.091 | 0.227 |
| Besancon | France | Native | 1 | 3 | 0.087 | 0.043 | 0.087 | 0.272 |
| Shingle | New Zealand | Introduced | 1 | 3 | 0.007 | 0.004 | 0.007 | 0.937 |
| Meadowbank | New Zealand | Introduced | 2 | 3 | 0.008 | 0.005 | 0.006 | 0.946 |
| Toklumen | Turkey | Introduced | 10 | 3 | 0.007 | 0.004 | 0.005 | 0.961 |
| Vodnany_Hluboka | Czech Republic | Farmed | 5 | 4 | 0.121 | 0.107 | 0.120 | -0.013 |
| Vodnany_Tabor_new | Czech Republic | Farmed | 4 | 4 | 0.115 | 0.102 | 0.117 | 0.009 |
| Vodnany_Tabor_old | Czech Republic | Farmed | 5 | 4 | 0.127 | 0.101 | 0.112 | 0.050 |
| LeLangan | France | Native | 3 | 4 | 0.070 | 0.058 | 0.071 | 0.401 |
| Cascina | Italy | Native | 3 | 4 | 0.042 | 0.034 | 0.042 | 0.647 |
| Gola | Ukraine | Native | 7 | 4 | 0.010 | 0.008 | 0.009 | 0.923 |
| Gedikli | Turkey | Introduced | 12 | 4 | 0.006 | 0.005 | 0.005 | 0.960 |
| Kirikkale | Turkey | Introduced | 16 | 4 | 0.007 | 0.004 | 0.005 | 0.961 |
| Korgalzhyn | Kazakhstan | Native | 3 | 4 | 0.005 | 0.003 | 0.004 | 0.967 |
| Vodnany_Velke | Czech Republic | Farmed | 4 | 5 | 0.101 | 0.096 | 0.112 | 0.049 |
| VegasdelGuadiana | Spain | Farmed | 10 | 5 | 0.004 | 0.002 | 0.002 | 0.979 |
| Vodnany_Madar | Hungaria | Farmed | 11 | 6 | 0.090 | 0.083 | 0.088 | 0.259 |
| Vodnany_Hluboka_old | Czech Republic | Farmed | 5 | 6 | 0.089 | 0.072 | 0.080 | 0.324 |
| Rosjon | Sweden | Native | 1 | 6 | 0.059 | 0.030 | 0.059 | 0.480 |
| Galkhyttedammen | Sweden | Native | 1 | 6 | 0.043 | 0.022 | 0.043 | 0.610 |
| Pend'Oreille_Lone | United States | Introduced | 3 | 6 | 0.010 | 0.006 | 0.009 | 0.923 |
| Prosser | Australia | Introduced | 3 | 6 | 0.008 | 0.005 | 0.007 | 0.942 |
| Dlhe_luky | Slovakia | Native | 1 | 7 | 0.089 | 0.045 | 0.089 | 0.189 |
| Felchowsee | Germany | Native | 6 | 7 | 0.090 | 0.084 | 0.092 | 0.220 |
| Vodnany_Vodnany | Czech Republic | Farmed | 5 | 7 | 0.094 | 0.079 | 0.088 | 0.255 |
| Mionnay | France | Farmed | 10 | 7 | 0.084 | 0.081 | 0.086 | 0.275 |
| Belley | France | Native | 4 | 7 | 0.061 | 0.053 | 0.061 | 0.480 |
| Kovada | Turkey | Introduced | 17 | 7 | 0.006 | 0.004 | 0.004 | 0.968 |
| Lijiang | China | Introduced | 3 | 8 | 0.117 | 0.094 | 0.116 | 0.017 |
| Kurowo | Poland | Native | 7 | 9 | 0.114 | 0.110 | 0.119 | -0.006 |
| Zabieniec | Poland | Farmed | 5 | 9 | 0.107 | 0.095 | 0.106 | 0.100 |
| Perugia | Italy | Introduced | 9 | 9 | 0.033 | 0.079 | 0.084 | 0.707 |
| Silver | United States | Introduced | 4 | 9 | 0.009 | 0.006 | 0.008 | 0.928 |
| Kizil | Turkey | Introduced | 5 | 9 | 0.009 | 0.007 | 0.008 | 0.935 |
| Heritage | United States | Introduced | 3 | 10 | 0.010 | 0.006 | 0.009 | 0.919 |
| Clyde | Australia | Introduced | 15 | 10 | 0.007 | 0.005 | 0.005 | 0.957 |
| Barilla | Australia | Introduced | 16 | 10 | 0.006 | 0.004 | 0.005 | 0.961 |
| Vodnany_Rumunsko | Romania | Farmed | 9 | 11 | 0.120 | 0.101 | 0.107 | 0.096 |
| Oborin | Slovakia | Native | 2 | 11 | 0.051 | 0.034 | 0.046 | 0.613 |
| Tulcea | Romania | Native | 5 | 11 | 0.019 | 0.017 | 0.019 | 0.840 |
| Vodnany_Kozak | Czech Republic | Farmed | 4 | 12 | 0.100 | 0.084 | 0.098 | 0.175 |
| Bonferraro | Italy | Farmed | 3 | 12 | 0.044 | 0.035 | 0.043 | 0.635 |
| Plovdiv | Bulgaria | Farmed | 2 | 12 | 0.031 | 0.020 | 0.030 | 0.748 |
| Gilette | United States | Introduced | 5 | 12 | 0.010 | 0.007 | 0.009 | 0.921 |
| Jordan | Australia | Introduced | 15 | 12 | 0.007 | 0.005 | 0.005 | 0.954 |
| Kleiner_Dollnsee | Germany | Native | 6 | 13 | 0.081 | 0.072 | 0.079 | 0.334 |
| Vendee | France | Native | 4 | 13 | 0.065 | 0.055 | 0.070 | 0.403 |
| Ili | Kazakhstan | Introduced | 3 | 14 | 0.006 | 0.003 | 0.004 | 0.963 |
| Sprague | United States | Introduced | 7 | 15 | 0.008 | 0.007 | 0.008 | 0.935 |
| Hamilton | New Zealand | Introduced | 11 | 17 | 0.082 | 0.081 | 0.086 | 0.275 |
| Stillingfleet | England | Native | 11 | 18 | 0.145 | 0.106 | 0.111 | 0.058 |
| Warbutts | England | Native | 8 | 18 | 0.112 | 0.090 | 0.097 | 0.182 |
| StLaw | Canada | Introduced | 16 | 19 | 0.089 | 0.087 | 0.090 | 0.236 |
| Ceres | South Africa | Introduced | 3 | 19 | 0.019 | 0.013 | 0.017 | 0.854 |
| Vodnany_Konigswartha | Germany | Farmed | 10 | 21 | 0.083 | 0.076 | 0.080 | 0.320 |
| Stolac | Bosnia | Introduced | 5 | 21 | 0.061 | 0.054 | 0.061 | 0.481 |
| Bouligneux | France | Farmed | 25 | 24 | 0.092 | 0.090 | 0.092 | 0.226 |
| Zurich | Switzerland | Native | 13 | 25 | 0.075 | 0.075 | 0.078 | 0.341 |
| Hessen | Germany | Native | 16 | 26 | 0.071 | 0.061 | 0.064 | 0.462 |
| Orangeville | Canada | Introduced | 10 | 27 | 0.110 | 0.100 | 0.106 | 0.102 |
| Blagoevgrad | Bulgaria | Introduced | 5 | 27 | 0.056 | 0.068 | 0.076 | 0.353 |
| Siverskyj | Ukraine | Native | 8 | 27 | 0.008 | 0.006 | 0.006 | 0.946 |
| PendOreille | United States | Introduced | 19 | 28 | 0.010 | 0.009 | 0.009 | 0.925 |
| SatopySamulewo | Poland | Native | 11 | 29 | 0.084 | 0.081 | 0.085 | 0.282 |
| Haaven | Germany | Native | 10 | 29 | 0.082 | 0.077 | 0.081 | 0.312 |
| Kuehrener | Germany | Native | 15 | 29 | 0.083 | 0.076 | 0.079 | 0.331 |
| Ceresole | Italy | Farmed | 10 | 30 | 0.040 | 0.039 | 0.041 | 0.651 |
| Volga | Russia | Native | 15 | 37 | 0.025 | 0.023 | 0.024 | 0.794 |
| Emajogi | Estonia | Native | 20 | 38 | 0.042 | 0.038 | 0.039 | 0.667 |
| Linkebeek | Belgium | Native | 16 | 46 | 0.106 | 0.105 | 0.109 | 0.081 |
| Lugano | Switzerland | Native | 18 | 52 | 0.036 | 0.035 | 0.036 | 0.692 |
| Sapanca | Turkey | Introduced | 7 | 60 | 0.014 | 0.011 | 0.012 | 0.898 |
| Ghazian | Iran | Native | 8 | 73 | 0.116 | 0.131 | 0.142 | 0.141 |
| Osikovica | Bulgaria | Native | 10 | 85 | 0.038 | 0.033 | 0.035 | 0.702 |
